# Is migraine a common manifestation of CADASIL? Arguments Pros

**DOI:** 10.1186/s10194-025-01980-x

**Published:** 2025-04-01

**Authors:** Hugues Chabriat

**Affiliations:** 1 Centre Neurovasculaire Translationnel-CERVCO - Département de Neurologie, APHP and Université Paris- Cité, Paris, France; 2 INSERM U1127, Paris Brain Institute, 75013 Paris, France

## Abstract

**Background:**

Migraine with aura (MA) is a hallmark feature of CADASIL, a hereditary small-vessel disease caused by NOTCH3 mutations. While MA is prevalent in CADASIL, its underlying mechanisms remain unclear, and the links observed can be questioned or debated. This study examined the prevalence, clinical characteristics, and pathophysiology of MA in patients with CADASIL.

**Methods:**

Clinical and experimental data were reviewed to assess MA prevalence, aura characteristics, sex differences, and pathophysiological insights from CADASIL models to confirm the indisputable pathophysiological links between migraine and aura and this unique genetic model of cerebral small vessel disease.

**Results:**

MA was 10–20 times more prevalent in patients with CADASIL than in the general population, with frequent atypical and prolonged auras. The altered sex distribution and delayed onset suggest disease-specific mechanisms. Experimental data also revealed heightened susceptibility to cortical spreading depression (CSD) in preclinical CADASIL models, linked to greater fragility in maintaining cortical ionic homeostasis.

**Conclusion:**

The high prevalence and distinct MA features, as well as the data obtained at the preclinical level, strongly support a causal relationship mediated by neurovascular dysfunction in CADASIL. Accumulating data in this condition sheds new light on the much-debated relationship between migraine and cerebrovascular diseases.

## Introduction

Migraine with aura corresponds to recurrent episodes of headache preceded or accompanied by transient focal neurological symptoms [1]. The International Classification of Headache Disorders (ICHD) diagnostic criteria for migraine with aura (MA) require at least two attacks with specific aura symptoms (visual, sensory, speech/language, motor, brainstem, or retinal) and at least three of the six defined characteristics including gradual onset, succession of symptoms, duration, unilaterality, positive symptoms, and association with headache [1]. Consequently, the diagnosis of MA corresponds to a purely clinical syndrome comprising transient neurological manifestations that develop according to a specific chronological and topographical pattern [2]. These manifestations reflect transient alterations in cortical function, which have been repeatedly reported in humans, both in the absence and presence of various conditions.

Cerebral Autosomal Dominant Arteriopathy with Subcortical Infarcts and Leukoencephalopathy (CADASIL) is a rare and specific condition and is now considered the most common hereditary cerebral small vessel disease (cSVD) worldwide [3]. The disease is caused by cysteine mutations in the NOTCH3 gene, leading to a progressive accumulation of extracellular domains (ECD) of the NOTCH3 protein in the wall of cerebral arterioles and capillaries [4, 5]. This accumulation, which increases with aging, presumably results in early dysfunction and subsequent structural and severe functional alterations of the cerebral microvessels [6–8]. In advanced stages of the disease, segmental loss of smooth muscle cells develops in the wall of arterioles [8, 9].

The potential relationship between MA and CADASIL has been raised since the initial discovery of the genetic disease in the 90 s, even before NOTCH3 gene identification [10]. After the spread of diagnostic genetic testing, MA was found rapidly as a prominent manifestation of the disease in multiple pedigrees [11, 12]. In another context, the nature of the association, which has been repeatedly confirmed between MA and Magnetic Resonance Imaging (MRI)-detected white matter hyperintensities (WMH), presumably related to sporadic cSVDs, remains unsolved [13–15]. Thus, a genetic model of cSVD represents a unique opportunity to explore the complex interplay between specific alterations occurring in the cerebral microvasculature and MA and to help select more targeted treatments for MA in this setting.

Since the discovery of CADASIL and the considerable advancement during the last decade of genetic diagnostic testing for different forms of monogenic cSVDs, substantial evidence has accumulated demonstrating a strong association between CADASIL and MA.

### The marked increase in the prevalence of migraine with aura in CADASIL patients is unlikely coincidental

In the general population, the prevalence of migraine, particularly MA, varies widely according to continent and study. The general prevalence of migraine is estimated to be approximately 11.4%, with 18% among females and 6.5% among males [16, 17]. Migraine with aura would represent 20–30% of all migraine cases, indicating that the prevalence of MA in the general population ranges from 2.3% to 3.4% in western countries [16, 17]. In contrast, a meta-analysis indicated that in Asia, the general prevalence of migraine would be only around 10.1%, and that the proportion of individuals with MA would be lower than that found in European migraineurs, between 10 and 15% [18]. This would correspond to a prevalence of MA varying between 1.0% and 1.5%, in line with the estimates obtained in Asia for individuals suffering from chronic migraine [19].

From the first description of CADASIL, MA was detected extremely frequently among symptomatic family members and was initially considered a common phenotypic feature of the disease [20, 21]. In the first CADASIL families ever reported in the literature, the prevalence of MA varied among pedigrees. In some cases, MA was detected in more than three out of four symptomatic individuals and was considered the central manifestation of the disease [11, 12]. In a large cohort of patients from the UK, the prevalence of MA was estimated to be 67.7% among 300 symptomatic patients [22]. In another largely documented study of migraine among 378 patients from France, 54.5% of the patients presented with a positive history of migraine, mostly MA (84%), corresponding to a global prevalence of 45% [23]. These large datasets also showed that 10–15% of individuals with CADASIL suffered exclusively from migraine attacks without aura [23]. Altogether, these findings, with additional confirmatory data increasing over time [24–28] strongly support that the prevalence of MA is between 10 and 20 times higher in CADASIL patients than in the general population. This considerable increase could not be explained by chance alone.

### The high prevalence of MA in CADASIL is consistent across families, and sex affects MA occurrence differently

The high frequency of MA in CADASIL has not been reported in just one CADASIL family or in a group of families, but in many unrelated pedigrees in different Western countries [27, 29, 30]. These findings rule out potential geographical or environmental explanatory factors. Moreover, in a large sample of CADASIL patients, Guey et al. observed that approximately 63% of women and 46% of men suffered from MA attacks [23]. This limited sex contrast, later confirmed in a large CADASIL cohort from the UK [22], differed from what was observed in the general population. MA was nearly three times more frequent in women than in men in Europe, with respective estimates of 3.6 to 5.4% and 1.3 to 1.95% [31, 32]. Moreover, the peak age at onset of MA was found to be globally delayed in patients with CADASIL by more than a decade compared to that reported in the general population. It was also found earlier in women than in men, which is the opposite of what is usually observed outside this genetic context [22, 23, 33]. These results suggest that the effect of sex on the onset of MA is modified in the presence of CADASIL and confirm the presence of one or more factor(s) triggering migraine attacks with aura, distinct from or in addition to those involved in the general population.

### The marked increase of migraine with atypical aura represents an original feature of CADASIL

While atypical aura symptoms are rarely reported in the general population, 59.3% of CADASIL patients with MA already present with atypical auras [23]. One in five patients with MA had motor symptoms during their auras. One in five patients presented with various brainstem symptoms, such as alterations of consciousness from confusion to coma. The same percentage of patients experienced acute-onset auras and 6% experienced long-lasting auras [23, 34]. The prevalence of migraine aura without headache in patients with CADASIL was much higher than the estimated lifetime prevalence of 1.5% in the general Italian population [34].

Moreover, a sharp rise in complicated forms of MA has been observed in patients [35–38]. While the corresponding symptoms are exceptional in the general population, they have been reported in nearly 10% of patients in a large CADASIL cohort [22]. Such episodes are often preceded by usual aura manifestations but which are prolonged by symptoms as reduced consciousness (confusion or coma) with sometimes seizures, fever, hallucinations and meningism [22, 39–43]. It can last up to one–two weeks [39–43]. These highly specific clinical features observed in a vessel disease such as CADASIL perfectly mimic those detected in a genetic and archetypal form of migraine, Familial Hemiplegic Migraine (FHM), which, on the other hand, is caused by mutations in genes that disrupt the functioning of ion channels in neurones or astrocytes [44–47]. In summary, there is a growing body of evidence that not only the occurrence of MA, but also its clinical expression is profoundly changed in the presence of the genetic cSVD. These clinical features are fairly specific and are not associated with sporadic forms of ischemic cSVD [48].

### Most clinical aspects of MA in CADASIL are compatible with experimental cortical spreading depression

The onset of aura symptoms in patients with CADASIL is most frequently progressive and develops initially with visual disturbances, followed sometimes by sensory disturbances, and, less frequently, in the most severe cases, by subsequent motor or aphasic symptoms [23]. These features have been repeatedly reported during classical auras [1] and are in accordance with the progression of neuronal depolarization at the cortical level and from back to front, as described during experimental cortical spreading depression (CSD) [49].

Although a huge increase in the prevalence of MA is detected in CADASIL, in both males and females, the earlier onset of MA in women with CADASIL than in men suggests some persisting hormonal influence on the occurrence of aura symptoms. Experimentally, modifications in the threshold of potassium concentration to elicit CSD have been observed according to the estrous cycle and hormonal status in non-specific mouse models [50]. Hormonal effects on the threshold to elicit experimental CSD have also been demonstrated in mouse models of FHM [51].

In addition, age has a major effect on the occurrence of MA attacks in patients with CADASIL. The peak of onset is between 20 and 40 years, with a strong decrease in the frequency of attacks according to age [23, 30, 52]. Such negative effects of aging on CSD have been documented in experimental models [50].

The type of aura symptoms appears to be related to cortex morphology in patients with CADASIL. Jouvent et al. previously found that CADASIL patients who presented with visual symptoms during their MA had different asymmetry indices and width of their calcarine sulci with a smaller cortical thickness than those with other types of initial aura symptoms [53]. These findings are in line with experiments showing that while cortical depression waves can spread radially outwards from the stimulation area in lissencephalic brains, the curvature of the cortex in gyrencephalic brains, as in humans, can dramatically modify the diffusion of potassium and inhibit or facilitate expansion or CSD [54].

Even the severe cerebral manifestations observed during some attacks of MA, and the occurrence of cortical edema in the most severe cases, are compatible with histological changes during experimental CSD [36, 55–57]. Such findings mimic the clinical features reported in FHM, where the opening of the blood–brain barrier has already been documented in vivo [58]. Experimentally, repeated or prolonged CSD can alter blood–brain barrier (BBB) permeability by activating brain Matrix Metalloproteinases (MMPs), which can last several hours, in line with what is observed in encephalitic forms of MA in CADASIL [59]. Such transient structural changes can also lead to plasma protein leakage and cerebral oedema, as detected both experimentally in different contexts and clinically in CADASIL [60].

### Cortical spreading depression, the presumed physiological substrate of migraine aura, is enhanced in the CADASIL mouse model

In line with the high prevalence of MA in patients with CADASIL, Eikermann-Haerter et al. previously showed that the electrical threshold for CSD was approximately ten-fold lower in mice harboring a typical mutation of the NOTCH3 gene (TgNotch3R90C) than in wild-type mice [61]. Increased CSD susceptibility was also detected in the same mice that developed approximately 40% more CSDs during K^+^Cl^−^ application, which was also the fastest compared to CSD assessed in WT mice [62]. In agreement with clinical findings in CADASIL patients, female TgNotch3R90C mice also tended to exhibit a lower CSD threshold than males [63]. Interestingly, this increased susceptibility could develop in the total absence of cerebral lesions in the mouse model, as also reported in young patients who had repeated MA attacks, even with hemiplegic aura, in the total absence of white matter lesions [64]. In line, in adult CADASIL patients, no link has been established between the extent of WMH and MA [65]. Furthermore, MA decreases dramatically with age, in contrast to the progressive accumulation of cerebral ischaemic lesions over time. This discrepancy, as well as the onset of attacks that can occur long before the onset of cerebral lesions, support the idea that the underlying pathophysiological mechanisms are unrelated to cerebral ischaemia [66].

Accumulating data in CADASIL mouse models suggest a possible disruption in ionic homeostasis, particularly related to potassium (K^+^) imbalance at the cortex level [67]. This was detected in the presence of NOTCH3 gR90C or R169C mutations in animals with middle cerebral artery occlusion [67] and was presumably related to a defect in extracellular K + clearance [67]. Additional data suggest that some astrocytic functions are largely impaired, with major functional or structural changes in the endfeet in CADASIL [68]. Modifications in the distribution of aquaporin channels have been also detected in the astrocytic feet of patients [68]. These channels play a key role in the regulation of extracellular space K^+^ concentrations and are involved in CSD [69, 70]. These astrocytic endfeet, which surround blood vessels and neurones, help buffer K^+^ by uptaking it through specific potassium channels, primarily Kir4.1 (which rectifies inward channels) [71]. A plausible hypothesis is that functional, structural, or numerical changes in ionic channels, modified in contact with Notch3-ECD, could be involved. Interestingly, Dabertrand et al. observed a depletion of Kir2.1 channels of capillaries in a CADASIL mouse model, which was related to a reduction in phosphatidylinositol 4.5-biphophonate (PIP2) [72]. Whether a similar mechanism could be involved in astrocytic Kir4.1 channels needs further investigation.

## Conclusion

The link between MA (MA) and CADASIL is robust, with a markedly increased MA prevalence in CADASIL patients, up to 20 times higher than that in the general population. This strong association holds consistently across multiple geographically diverse CADASIL families, indicating that it is not environmentally dependent. MA attacks in CADASIL have relatively specific features, such as atypical and complicated forms of auras, which have never been observed in sporadic cSVD. The delayed age of onset also underscores the potential dose–effect of NOTCH3 accumulation before the occurrence of attacks, although exceptional cases are possible in young children. Further investigations are needed in preclinical models to clarify whether a particular threshold of NOTCH3 protein accumulation promotes cortical spreading depression. This temporal pattern is influenced by age and sex. Biological plausibility is established through CADASIL-related vascular and ionic dysfunction, which aligns with well-known MA mechanisms. Different MA features in patients with CADASIL are also similar to those reported in FHM, where ion-channel dysfunctions are associated with MA, providing a comparative basis for understanding MA in genetic cSVD. Finally, there is a large coherence supported by CADASIL models, demonstrating lower CSD thresholds, the most likely aura mechanism.

All these elements compellingly argue that CADASIL significantly elevates MA risk through a unique pathophysiological pathway and that the link between CADASIL and MA is actually causal.

## Response to Wang YF [73]. The Journal of Headache and Pain

### Response

My colleague, Dr Wang, claims that ‘Migrainous headaches in CADASIL are not equal to a migraine diagnosis’ and that migraine is a “specific diagnosis rather than just a symptom”. He also states, “migrainous headaches should be coded as ‘headache attributed to Cerebral Autosomal Dominant Arteriopathy with Subcortical Infarcts and Leukoencephalopathy (CADASIL)’ (code 6.8.1) rather than migraine with or without aura (codes 1.1 and 1.2) according to the ICHD-3” [74]. I disagree with this diagnostic approach. Just because we have diagnostic rules does not mean that the question of migraine with aura can no longer be raised in CADASIL. Even taking part in the present debate would be pointless if migraine or migraine with aura could not be considered in the presence of any vascular pathology.

In reality, diagnostic tools such as the ICHD-3 classification are no more than a collection of information that enables us to find our way at a given time within the nosological framework of headache disorders [75]. This classification is particularly useful for the diagnosis of headache, since the latter is based almost entirely on the record of clinical information from a detailed interview of the patient. However, the Headache diagnostic criteria should only be considered as benchmarks that help researchers and clinicians to discuss a common language [76]. These tools do not define pure and unique pathophysiological entities. Thus, the ICHD-3 diagnostic criteria must not be locked into distinct and definite ‘diseases’ that cannot be questioned. The results of the experiments in the field should also be considered. They are particularly enlightening in this regard. For example, spreading depression, which is likely the basis of migraine with aura, corresponds to a physiological phenomenon that can be observed in any nervous tissue [77, 78]. Conversely, the factors and conditions favouring such a phenomenon can vary considerably [15]. Age [79], sex [80], hormonal impregnation [80], level of cerebral perfusion [81], micro-emboli [66, 82], mechanical stimuli [83], local ionic variations [50, 84] dysfunction of ion channels of genetic origin [69], are all circumstances that can promote, more or less, the occurrence of depolarization waves at the cortex level as first described by Leao [85]. In clinical practice, one of these factors or their combination is also commonly involved in the occurrence of migraine attacks with aura in patients [86]. Therefore, I think that confining the entity ‘migraine with aura’ to a nosologically closed category is a mistake if we are to consider all our knowledge and the most recent scientific advances. I think that the real difficulty here lies mainly in the misconception of migraine or migraine with aura as a disease rather than as a syndrome. To make progress, I strongly believe that migraine with aura should be considered a syndrome, most likely related to the occurrence of cortical spreading depression. The latter can be initiated in multiple circumstances and in various contexts or diseases. With the discovery of CADASIL, we learned that some structural or functional alterations at the microvascular level that can extend to capillaries could also promote the occurrence of migraine attacks with aura [23, 87]. If we consider, as my colleague does, that migraine only corresponds to a specific category, the terms migraine or migraine with aura should be used only for primary headaches. If we were to consider the case of epilepsy in the mirror, this would be tantamount to saying that a tumour cannot be the cause of epilepsy, and that the term “epilepsy” should only be used in the absence of focal lesions. Clearly, this finding is neither valid nor consistent with our clinical practice.

My colleague states in his report that “it is not uncommon that migraine symptoms in CADASIL may not conform to the classical manifestations of “migraine aura”, and could not easily fulfil the diagnostic criteria for migraine with aura”. Interestingly, this argument of “atypicality” of aura symptoms, which I used as a support for a specific clinical expression of the vascular disease, is completely overturned. However, this argument cannot hold if we consider another category of migraine, familial hemiplegic migraine (FHM) [88], which is also defined in the current ICDH-3 diagnostic classification. These pure migraine disorders, classified as primary types of headaches, are caused by channel dysfunction of neurones or astrocytes, which can promote changes in ionic concentrations or glutamate release at the cortical level [89, 90], facilitating the occurrence of spreading depression. These genetic disorders are responsible for migraine attacks with prolonged aura, motor deficits, encephalopathy, and even coma, which are atypical features reported in CADASIL patients [47, 91, 92]. Thus, frequent attacks with atypical patterns of auras may occur with a reduced threshold for the occurrence of spreading depression, regardless of the exact origin of this reduction, vascular or not, and even for migraine categories with an indisputable diagnosis.

Dr Wang also writes, “As patients with CADASIL could have endothelial dysfunction that predisposes the patients to increased risks for cerebral ischemia, whether some of the “auras” could be symptoms of transient ischemic attacks or even minor stroke in patients with a history of headache awaits further confirmation.” I think such a hypothesis is not supported today, and conversely, accumulating data establish that migraine with aura in CADASIL is not related to cerebral ischaemia. In a PET study of patients with CADASIL, migraine attacks with aura were detected in several individuals younger than 30 years whose cerebral blood flow, particularly cortical blood flow, was higher than that in the control population [93]. There is also a clear misalignment between the onset of migraine with aura at an early stage of the disease (sometimes during childhood) and the late appearance of ischaemic lesions or stroke events, detected several decades after the onset of attacks of migraine with aura or even after their total disappearance [21, 23]. Furthermore, the occurrence of migraine with aura was not linked to the accumulation of cerebral ischaemic lesions or clinical manifestations. Recent results show exactly the opposite; patients with migraine with aura present with less ischaemic stroke after adjustment for age and vascular risk factors (personal data) [23]. In parallel, propagating depression waves induced by KCl application were facilitated at an early stage in TgNotch3R90C mutant mice, which was not observed in wild-type mice with chronic hypoperfusion [62]. Finally, no corresponding focal ischaemic lesion was observed in the cortex of CADASIL patients with migraine and aura. Moreover, there is no reason why auras usually begin with visual disturbances in CADASIL [23, 94], as observed classically in migraine with aura, whereas cerebral microvasculature is diffusely affected by the disease [95].

My opponent argues that individuals with the p.R544C variant, a specific cysteine variant of the NOTCH3 gene associated with CADASIL in East Asia, have been repeatedly detected in a large number of individuals whose clinical manifestations did not include migraine with aura, in contrast to the phenotype of Caucasian families with other mutations. This is true [96–98]. However, the initial vision of a unique phenotype in carriers of a pathogenic cysteine mutation in NOTCH3 has been strongly challenged. The location of the mutation within the EGFR domain of the NOTCH3 gene was recently shown to have a major influence on the accumulation of the NOTCH3 protein in the vessel wall, as well as on disease severity [99–101]. The migraine component might also be modulated in the phenotype according to different genetic variants, as detected with another phenotypic trait, the development of white matter hyperintensities in the temporal lobes, which is an exceptional feature in the presence of the R544C variant [98]. A possible interaction with other genetic factors linked to ethnicity cannot be excluded either, in line with the low prevalence of migraine and migraine with aura in Asian patients with CADASIL, as well as in the general population [102]. Additional studies on the prevalence of migraine with aura according to NOTCH3 mutation location in Asian CADASIL populations are still needed. However, the exact underlying reasons for this variability remain undetermined. Additional investigations at the preclinical level may help to explore the influence of diverse NOTCH3 variants on the spreading depression trigger threshold and decipher the underlying biological mechanisms.

Dr Wang finally claims, “an interesting study showed that there was no difference in Calcitonin Gene-related Peptide (CGRP) levels between CADASIL patients with and without migraine”. CGRP is one of the few markers released into the peripheral circulation during headache attacks involving the trigeminovascular system [88]. It clearly increases during migraine attacks. However, no increase is usually detected outside migraine attacks, except in patients with chronic migraine [103]. In the negative study cited by Dr. Wang, measurements were taken in patients with CADASIL outside of migraine attacks, which cannot lead to any conclusion. I also strongly believe that the negativity of a dosage, which has its own limitations and can vary according to many factors and technical conditions, cannot be used as a solid argument to reject clinical facts that are undeniable, particularly when the diagnosis is purely clinical. Finally, I think that his additional arguments related to the lack of difference in the prevalence of migraine with aura between individuals carrying a NOTCH3 gene variant and those who do not; in large population-based studies must be used with infinite caution [104]. In these large studies, the diagnosis of migraine was based on crude tools, such as questionnaires, and the occurrence of rare or old attacks of migraine with aura can lead to major recall biases, particularly in middle-aged and older individuals. The results also depend on the quality of clinical questioning and level of expertise in migraine diagnosis. These aspects are key to considering and are often neglected. I believe that this type of data with diagnoses derived from self-questionnaires is not sufficiently solid to draw any conclusions.

In summary, I did not find Dr. Wang's comments convincing enough to undermine the established link between migraine with aura and CADASIL.

## Data Availability

No datasets were generated or analysed during the current study.
